# Motives and preferences of general practitioners for new collaboration models with medical specialists: a qualitative study

**DOI:** 10.1186/1472-6963-7-4

**Published:** 2007-01-05

**Authors:** Annette J Berendsen, Wim HGM Benneker, Betty Meyboom-de Jong, Niek S Klazinga, Jan Schuling

**Affiliations:** 1 Department of General Practice, University Medical Centre Groningen, University of Groningen, Antonius Deusinglaan 1, 9713 AV Groningen, The Netherlands; 2 Department of Social Medicine, Academic Medical Centre, University of Amsterdam, The Netherlands

## Abstract

**Background:**

Collaboration between general practitioners (GPs) and specialists has been the focus of many collaborative care projects during the past decade. Unfortunately, quite a number of these projects failed. This raises the question of what motivates GPs to initiate and continue participating with medical specialists in new collaborative care models.

The following two questions are addressed in this study:

What motivates GPs to initiate and sustain new models for collaborating with medical specialists?

What kind of new collaboration models do GPs suggest?

**Methods:**

A qualitative study design was used. Starting in 2003 and finishing in 2005, we conducted semi-structured interviews with a purposive sample of 21 Dutch GPs. The sampling criteria were age, gender, type of practice, and practice site. The interviews were recorded, fully transcribed, and analysed by two researchers working independently. The resulting motivational factors and preferences were grouped into categories.

**Results:**

'Developing personal relationships' and 'gaining mutual respect' appeared to dominate when the motivational factors were considered. Besides developing personal relationships with specialists, the GPs were also interested in familiarizing specialists with the competencies attached to the profession of family medicine. Additionally, they were eager to increase their medical knowledge to the benefit of their patients. The GPs stated a variety of preferences with respect to the design of new models of collaboration.

**Conclusion:**

Developing personal relationships with specialists appeared to be one of the dominant motives for increased collaboration. Once the relationships have been formed, an informal network with occasional professional contact seemed sufficient.

Although GPs are interested in increasing their knowledge, once they have reached a certain level of expertise, they shift their focus to another specialty.

The preferences for new collaboration models are diverse. A possible explanation for the differences in the preferences is that professionals are more knowledge driven than organisation driven as the acquiring of new knowledge is considered more important than the route by which this is achieved. A new collaboration model seems a way to acquire knowledge. Once this is achieved the importance of a model possibly diminishes, whereas the professional relationships last.

## Background

Physicians appear to attach much value to their professional autonomy. The distinguishing characteristic of professionalism is that professionals themselves control the access to the profession and determine the required knowledge and skills to practise [1,2]. It is therefore important to match collaboration models between professionals with the professionals' interests. Besides, the way GPs and specialists interact has important implications in health care systems where the GP is the gatekeeper to specialist care.

New collaboration models are assumed to improve the efficiency of patient care and to contribute to decreasing costs, particularly in cases of chronic illness. During the past decade, in Great Britain and in The Netherlands, experience has been gained in the development and organization of this type of care. The barriers to the integration of care are almost the same in these two countries [3]. They include structure, procedures, finance and legitimacy at system and institutional level as well as the professional self-interest at the operational level. The professional barriers were: -competing ideologies and values; -professional self-interest and autonomy, and inter-professional competition for domains; -conflicting views about patients' interests and roles. Changes are necessary in the manner in which physicians carry out their professional duties and how they perceive their role in the medical profession [4,5].

In recent years *quantitative *methods were used to examine the collaboration between GPs and specialists [6-9]. *Qualitative *studies in this area were confined to reporting on special topics such as the prescribing of specialist medication [10], collaborative care for patients with rheumatism [11], the implementation of evidence based medicine [12], integrated care for asthmatic patients [13], and hospital at home care [14,15]. Although GPs' opinions of collaboration were surveyed in the late nineteen-nineties, those studies concentrated on the relationships between GPs and specialists without a specific focus on new collaboration models [16-18].

The implementation of changes seems to depend for an important part on professionals working collaboratively. This collaboration is necessary both to develop and initiate new forms of collaboration and to implement them in the regular care setting. In another study we examined the opinions and preferences of medical specialists [19].

In this study the following research questions were investigated:

What motivates GPs to initiate and sustain new models for collaborating with medical specialists?

What kinds of new models of collaborative practice do GPs suggest?

## Methods

We chose to use an exploratory qualitative research design, as little is known about the motives and preferences of GPs with respect to the development of new collaborative practice models. We defined new collaboration models as any kind of collaboration other than conventional contact by telephone or letter about a patient. As personal motives play an important role in this area, we chose a format consisting of semi-structured interviews to gain as many personal insights as possible. Ethical approval was not required.

### Study population

We selected a purposive sample of 21 Dutch GPs in order to obtain a variety of opinions and experiences. Sampling was based on the following factors: gender (14 men, 7 women), age (29 to 61 years), location of practice (13 urban, 8 rural), type of practice (15 with a group versus 6 solo; with or without pharmacy services), academic involvement in post-graduate education or professional committees, and experience with new models of collaboration (whether or not they were successful).

### Data gathering

The interviews were conducted by two GPs, each trained and experienced in conducting interviews. In earlier research, it was reported that the advantages of a medical colleague conducting the interview outweighed the disadvantages of having a lay person conduct the interviews. These advantages include improved access, better understanding of the issues, and the ability to challenge the interviewees [16].

The goal of the research was explained prior to each interview. Subjects were also told that anonymity would be preserved during analysis of the data. The focus of the interview was the professional role of the GP. The main topics for discussion were:

• positive and negative experiences with medical specialists

• whether or not GPs were willing to participate in the initiation of new models of collaboration

• what form such collaboration should take

• to which patient groups it would apply.

The subjects were asked to use concrete examples to illustrate their opinions. The questions did not have to follow a set order to allow the subjects to freely associate among different topical areas.

### Analysis

Each interview was recorded on audiotape and transcribed verbatim. Working independently, three researchers (two GPs and a medical student) assigned labels to what they felt to be the most important statements in the complete transcripts. Implicit as well as explicit statements were analyzed. The researchers then discussed any discrepancies in the findings until a consensus was reached. The transcripts were also read by a senior researcher to control for short, open and neutral questions.

The analysis was performed according to the rules for qualitative research and the framework method [20,21]. The five most important steps were familiarization, identifying a thematic framework, indexing, charting, and mapping/interpretation. The interviews and the analyses were conducted simultaneously. Kwalitan 5.0 software was used to process the information. The identified motives and barriers were categorized. The results are presented in table 1.

**Table 1:** Motives and barriers for collaboration

**Motives extracted from regular care experiences**	Patients' interest Developing personal relationships Gaining mutual respect (status)
**Motives for the development of new models**	Increasing medical knowledge of the GP Improving medical specialists' knowledge of the competencies of GPs Reciprocal inspiration
**Impediments to the development of new models**	Current level of collaboration is excellent Other priorities (private or work related) Negative experiences with new models
	• lack of thorough preparation
	• organizational aspects too demanding
	• prevalence of chosen patient categories too low
	• agreements are not kept
	• cynical attitude specialists
	• one way communication
	• lack of funding after project phase

## Results

All 21 GPs who were approached agreed to participate. The interviews were conducted between May 2003 and March 2005 inclusive and each lasted approximately one hour, although some lasted up to 2.5 hours.

A preliminary categorization of motives and preferences took place after four interviews and once more after thirteen. No new issues emerged after the thirteenth interview (saturation). The remaining interviews were used to validate the chosen categories and to provide additional illustrative examples.

### Motives for collaboration

All the GPs indicated that shared responsibility and care for the patient are essential elements of the collaborative relationship with the medical specialist. Extending the degree of collaboration would serve the patient's best interests, particularly for seriously ill patients and the elderly. An increased degree of collaboration would also speed up the referral process and facilitate the communicative pathways between the GP and the medical specialist.

#### Experiences from the regular care setting which may affect the development of new collaboration models

##### Personal relationships

Most of the interviewees indicated that collaboration is facilitated when the physicians involved know each other on a more personal level. This makes collaboration more enjoyable, more candid, and easier. A number of GPs stated that they are more inclined to use the telephone to discuss patient related issues when they are acquainted with the specialist involved. Additionally, they said that knowing each other personally also leads to a better understanding of each other's working method. The relevant medical competencies are better understood and there is increased insight in physician's behaviour with respect to the patient. This helps the GP choose the appropriate specialist for a specific patient.

"Knowing each other personally is what makes or breaks successful collaboration with specialists."

"You know how they are, how they treat their patients. It's important to have some idea of their professional knowledge, and they should have some idea of what a GP does."

##### Perception and status

GPs generally want to be regarded as competent colleagues by the specialists who take the patient's environment into account and who do not make unnecessary referrals. Many GPs would like to be accorded the same level of respect that specialists show each other.

"That the specialist values the GP as an academically trained professional who has his own expert knowledge of patients and an expertise which reflects the generalistic outlook of the GP."

Almost all of the GPs did not claim to experience any difference in status with the specialists, although such a difference is sometimes perceived in society.

They simply also earn more [...] and people naturally regard them differently. But I disagree."

The older GPs noticed a clear difference between present day practices and the past. As they age they experience less difference in status because they become more self-confident and they perceive less arrogance on the part of the specialists. Some did state however, that residents were sometimes prone to displaying arrogance.

*"As the years go by, you attach less and less importance to status*.

"The arrogant specialists have all retired by now anyway."

Most of the respondents stated that they did not perceive a difference in status. Contrary to this claim, we did find indications of an implicitly stated difference in status in the transcripts. Many GPs look up to specialists. This was evident in the answers pertaining to perception as well as in the arguments for changing the collaborative process.

"So, if I'm walking down the hallway, and a specialist pats me on the shoulder, it just makes me feel good."

#### Positive factors for developing new models of collaboration

##### Increasing medical knowledge

All of the GPs considered the increase of medical knowledge as a reason for participating in new models of collaboration. They enjoyed being able to subsequently use this knowledge for the benefit of their patients. They considered evidence based knowledge an important source of knowledge next to knowledge gained from experience.

##### Understanding each other's methods of working and professional competencies

All the interviewees considered it important for the specialists to increase and improve their understanding of the GPs' working method and the competencies associated with the profession of family medicine. The specialist should gain a better understanding of the conditions under which GPs work, their basis for making decisions, and the way that time is factored into patient management. Additionally, specialists could be made aware of the non-medical circumstances of a patient that often play a role in treatment decisions.

"As resident, I have occasionally had thought: how can a GP have missed that in such an abdomen? You work in this nice little cubicle, under the bright examining lights, neat examining cot. And once in a while you are called out to someone's house, and they have a waterbed [...] you should try to do a proper examination of the abdomen in those conditions!"

Some GPs find it sensible to improve their understanding of the specialists' working methods but do not consider this a primary goal. They said that, after all, they have been acquainted with the hospital setting since their undergraduate training period.

##### Reciprocal inspiration

Many GPs indicated that they found it stimulating when new joint initiatives were developed and that they felt more energetic when such initiatives were conducted properly. This leads to a more pleasant working environment. A number of GPs expressed the opinion that sharing the responsibility for patient care gives them a good feeling.

"It is so inspiring, not so much to learn, but simply to experience the feeling, enthusiasm and zest for work."

#### Impediments to the development of new models of collaboration

The respondents gave three main reasons for not changing the current level of collaboration.

Some GPs felt no change was needed as they considered the present level of collaboration to be excellent. Another objection to the development of new collaborative practices was grounded in the time commitment that would be necessary. Personal and work related issues were seen as more important. Finally, some of the GPs claimed that they had had negative past experiences with new models of collaboration. For example, they felt that there had been too much of a rush during the initiation phase of some project without adequate preparation taking place. On the other hand, other collaboration projects were organized to such an extent that they were too demanding with overly extensive protocols requiring excessive paperwork.

"At the time, transmural care consisted of an office with all kinds of employees, a nurse who took care of discharging the patient from the hospital home with all manner of bells and whistles. Well, that's how it was presented. If I think back to that, I see red."

"Such a fancy professional – a Jack-of-all-trades and master of none – corrupting the issue and hiring an expensive consultation firm, these are all insurance premiums."

Some projects involved patient categories rarely seen in daily family practice, causing the protocol and any accompanying information to be misplaced and forgotten.

In some projects either the GPs or the specialists did not comply with the agreements that were made and it was not clear who was responsible. Some GPs reported negative experiences with specialists because of their cynical attitude.

Many interviewees stated that projects should be developed to meet a common need. Some initiatives appeared to stem only from specialist needs and seemed to address only those tasks that the specialists wanted to move out of the hospital setting.

"Then he's developed an entire form, and it was sent to all of us, and then I get very nervous, because that form has to be put somewhere, and of course it takes another two months before I see someone like that, and he then wants to go to a different hospital. Well, fine. Things don't work that way, so I think it's all nonsense really."

"And then you suddenly get a patient who brings with him a schedule from the hospital, and he says: 'Doctor, you have to come now, because that's what the protocol says.' And then I think to myself #$%^%$, why don't you go fly a kite?"

Most GPs did not find it important to be compensated during the developmental stages of a project, though they did feel compensation was justified once the collaborative project was up and running.

"If you take it seriously, there will be a price tag attached."

### Specific new models of collaboration

#### Preferences for and experiences with specific new collaboration models

A number of different models for new collaborative practices were suggested which are presented in table 2. There were five types that were frequently mentioned, and three that were mentioned less frequently. Each type of model identified had its share of supporters and opponents. Some GPs had already gained experience with new models of collaboration, usually in the form of a project.

**Table 2:** Suggested designs for new models of collaboration

**New models**	**Forms**	**Patient categories**
Joint consultation	Multidisciplinary consultation Joint consultation in GPs' practice	Complex patients Seriously ill patients Common medical problems
Common guidelines for care	Collaboration including specialized nurses	Chronic illnesses
Joint treatment guidelines	Joint drug formularies	Frequently prescribed medicines
Diagnostics	Direct access for GPs to: Endoscopie Ultra sound examinations	
Hospital care at home	GPs as a member of a hospital at home team	Seriously ill patients
Hospital care	Visiting hospitalized patients by their GP	Seriously ill patients Problematic situations
Integrated emergency care	GPs cooperatives integrated with the hospital emergency department	Emergencies (out-of-hours service)

#### Joint consultation

Some respondents felt that joint consultation would be particularly useful. On the other hand, there were a few GPs who reported having had negative experiences while attending multidisciplinary discussions concerning cancer patients. They felt that they were regarded more as observers than as useful participants despite the significant investment of time involved.

The respondents stated that joint consultation involving a group of GPs, their patients, and a specialist was realizable for psychiatric patients and patients with more common medical problems such as orthopaedic, dermatological, gynaecological, urological, cardiac, and pulmonary conditions.

*It was a kind of role reversal. Him with his expertise and me with mine. A real exchange took place*.

For seriously ill patients, such as cancer patients or geriatric patients with complex conditions, the option of a house call by the specialist was seen as desirable. However, complications were foreseen in the planning and organization of such a visit.

#### Common guidelines for care and collaboration with nurses

A number of GPs are in favour of producing a set of common guidelines to be used by GPs, specialists, and nurses when providing collaborative care to patients with chronic illnesses such as diabetes, rheumatoid arthritis, and cardiac and pulmonary diseases.

"I like delivering organized care and that gives clarity, you can do something with it."

"You must not want to arrange things on a higher professional level than necessary. In fact, you have to arrange things at the lowest possible qualified level."

A similar number of GPs did not agree. They were sceptical about successful implementation, unwilling to relinquish their authority, or reluctant to take responsibility for the necessary organization.

"It's possible to set rules for certain things, but you shouldn't regulate everything too strictly."

"Oh yeah, you're supposed to include that. It's in the protocol. Well, that may be, but we do it differently."

"I don't like the idea. It sounds more like practice management."

#### Joint treatment guidelines

Agreements about prescriptions (joint drug formularies) only involved ardent proponents or adversaries. Proponents consider the long term effects and notice that young GPs are more likely to comply with guidelines. Adversaries dislike the insufficient involvement of the GP or were unwilling to give up their prescribing privilege.

"One of the most important things we have to do, besides making up a set of regular guidelines."

"A GP works within a group, without back-up, then along comes a specialist with the latest research, and that has more of an influence."

"The specialist dictates how it should be done and we simply nod in acquiescence."

#### Diagnostics

Many GPs appreciate the current diagnostic screening capabilities available, but would like the opportunity to access the diagnostic tools usually used only by the specialists, such as gastroscopy and trans-vaginal sonography, without necessitating the involvement of the specialist. Those GPs who worked with tele-dermatology were positive about their experience. Some of the GPs would like to see an expansion in their capabilities to include scope examinations and ultra-sound for diagnosing cardiovascular disorders. Other GPs were less favourable about adopting these technologies, stating that they could always refer the patient if necessary.

#### Hospital care at home

Hospital care at home is considered a favourable development, but this care is highly dependent upon the individual circumstances of the patient. The involvement of the GP is often minimal, and the respondents stated that any increase in involvement would need to be accompanied by adequate compensation. Some GPs felt that the development of hospital at home care has only served to alleviate bed shortage problems in hospital.

#### Miscellaneous

GPs do not feel that becoming involved in hospital care is a viable option. The most that some of the respondents felt they could contribute was to visit seriously ill patients to raise their spirits or to mediate in particularly problematic cases.

A number of GPs approved of integrating primary care services with the emergency department of a hospital (out-of-hours service).

GPs would be able to develop individual Special Interests if a sufficient number of GPs worked together in a collaborative setting. The respondents said that this could strengthen the GP's position when collaborating with specialists.

## Discussion

### Key findings

Any new model of care should meet the needs of the GP as an autonomous professional, providing good care for his patients.

The development of personal relationships and gaining mutual respect appear to be important motives for developing new collaborative care models. Although we expected that doctors would find it less important to get to know one another, in view of the increase in part-time employment and sub-specializations in recent years, the GPs clearly identified this as a need. The establishment of personal relationships is still often considered to be fundamental for the development of improved communication, trust, and collaboration.

GPs are eager to increase their medical knowledge for the benefit of their patients by collaborating with medical specialists. All GPs would like to introduce medical specialists to the competencies necessary in general practice. This could also reduce the perceived difference in status that exists between GPs and medical specialists. Many GPs indicated that they experienced reciprocal inspiration through shared responsibility.

The perceived barriers for new models were: current level of collaboration is excellent, other priorities (private or work related), and, negative experiences with new models.

GPs felt the need for collaboration was especially true for complex and seriously ill patients, patients with chronic illnesses or patients with common medical problems. Opinions did vary on this, however. GPs stated many different preferences with respect to new forms of collaborative practice.

### Study limitations and strengths

The qualitative design was well suited to an investigation of this topic. The GPs generally felt encouraged and stimulated to speak their minds. It was striking that all of the GPs experienced the interview as positive; it allowed them to organize their ideas about this topic. The strengths of this study are the qualitative method that really captured motivational factors and it's the first of its kind in this field.

The researchers were aware and alert to the possibility of bias during the research. Everything possible was done to acknowledge or minimalize, the effect of possible bias on the interviews and on the interpretation. Additionally, the results were discussed with a wide range of people. We discovered, as was also found in the earlier study that the advantages amply made up for the possible disadvantages.^16^

As this is a qualitative study, the outcomes have to be regarded as inductive.

### Comparison with existing literature

The importance of the existence or the development of personal relationships between GPs and medical specialists has also been demonstrated by other studies [2,6,11,16].

GPs are eager to learn from specialists and that is one of the reasons they support the development of new collaborative practices. This observation has also been reported previously [12,13,17]. Studies in which the practice of joint consultations was investigated have shown that this collaboration results in more expertise, better clinical skills, less additional diagnostic screening, and fewer referrals without any apparent negative effects on patients [22-24]. However, these latter studies also reported saturation when the learning experience was over.

A certain amount of uncertainty is inherent to the profession of the GP. Their long term relationships with their patients enable them to get to know their patients better [4,25]. Specialists do not always sufficiently appreciate the benefit of this [10]. This partially explains why GPs would like to convey this to specialists. In this study, a trend toward a diminishing difference in the status of GPs versus that of specialists was seen. This has also been observed in other studies [18].

GPs are willing to make an extra effort for both the seriously ill and the elderly. Their involvement with these categories of patients was also observed in other studies [10,14].

Many concerns about new collaboration models have been reported before. An overly complicated model of care leads to too many meetings, too much paperwork, and, consequently, increased irritation [13]. On the other hand, when patient information and communication are adequate, the GPs find the increase in workload to be less relevant [26].

New models of collaborative practice should not be introduced solely at the hospital level. One study, which reported on this topic, stated that hospital doctors had 'tell" and "sell" approaches when asking GPs to do something. GPs preferred the "sell" approach [10]. New models of collaborative practice should meet the GPs' needs, and the GPs want to be involved in the development of such initiatives. Models of care do not need to be developed for diseases which are rarely seen in the primary care setting [10,11].

## Conclusion

The development of personal relationships appears to be one of the dominant motives for the initiation of new models of collaboration. Knowing one another on a personal level improves the quality of collaboration. Once the relationship has been developed, an informal network with irregular professional contacts seems to be sufficient.

During the past decades general practice has become professionalized to such an extent that GPs would like to educate the specialists with respect to the day to day competencies involved in a general practice. This came across as a strong motive and fits in with the fact that GPs are experiencing a reduction in the difference between their status and that of specialists.

GPs want to invest in increasing their medical expertise. This motive may be seen as not sustainable if collaboration occurs within a single discipline. Once they have acquired a certain expertise GPs are interested in learning about other specialties.

This lack of sustainable motives has consequences for the implementation of new models of collaboration. Taking into account the factors which were identified as impediments to collaboration, adequate organizational support, perhaps in the form of family practice nurses and nurse practitioners, and financial support are necessary for any new collaborative practice to succeed.

The GPs interviewed preferred different kinds of collaboration. One possible explanation for this diversity may lie in the fact that professionals are more knowledge driven than information driven as the acquisition of new knowledge is more important to the GP than the route by which this is achieved. The GPs see new collaborative practices as a way to increase their professional knowledge. Once this knowledge has been absorbed, the continued existence of the collaborative model might be threatened, whereas the professional relationships will last.

GPs would like the specialists to learn more about the care delivered by the general practitioner. The development of this needs to be stimulated. Specialists do not feel that they have anything to learn from the GPs [19]. Thus, this may best be implemented during the post-graduate training of all physicians in order to maximize the benefits of this socialization thereafter.

The preferences of the specialists must be taken into account for any new collaboration initiative to be successful. In our related study, specialists did not suggest many new collaboration models [19]. If specialists did so they instantly came up with barriers. The GPs' preferences varied widely with respect to new collaboration models and the patient groups that would be targeted by such models. Quantitative research is necessary to investigate the general applicability of these results. Based on the information obtained in the present study, however, the perceived merits of professionalism (motives) seem to dominate over the design of new collaboration models.

## Competing interests

The author(s) declare that they have no competing interests.

## Authors' contributions

All authors contributed to the design and the write-up of this study. AJB was responsible for the day-to-day management and produced the first draft of the manuscript. AJB and WHGMB conducted the interviews and analyzed them together with JS. All authors read and approved the final manuscript.

## Pre-publication history

The pre-publication history for this paper can be accessed here:


